# Greater presence of receptors for relaxin in the ligamentum teres of female infants who undergo open reduction for developmental dysplasia of the hip

**DOI:** 10.1186/s13018-021-02784-w

**Published:** 2021-10-18

**Authors:** Semih Ayanoğlu, Haluk Çabuk, Fatmagül Kuşku Çabuk, Kubilay Beng, Timur Yildirim, Süheyla Uyar Bozkurt

**Affiliations:** 1 Orthopedics and Traumatology Department, İstanbul Altınbaş University, Mahmutbey, Dilmenler St. No: 26, 34217 İstanbul, Turkey; 2 Orthopedics and Traumatology Department, İstinye University, İstanbul, Turkey; 3 Department of Medical Pathology, İstanbul Bakırköy Dr Sadi Konuk Training and Research Hospital, İstanbul, Turkey; 4 Baltalimani Bone Diseases Training and Research Center, University of Health Sciences Turkey, İstanbul, Turkey; 5 Department of Medical Pathology, Medical School, Marmara University, İstanbul, Turkey

## Abstract

**Background:**

While many factors involved in the etiology of developmental dysplasia of the hip (DDH), one of which is the hormone relaxin. Relaxin concentrations in patients with DDH may lead to pathodynamic changes during hip development by altering the physiological nature of the ligament, as well as by long-term exposure to relaxin during pregnancy. Our objective in this study was to determine the number of relaxin receptors in the ligamentum teres and their role in causing DDH.

**Methods:**

We identified 26 infants between birth and 3 years of age who had undergone open reduction for DDH between 2010 and 2012. 12 hips of 12 miss abortus fetus between 20 to 35 weeks of gestation were used as control group. Specimens obtained from two groups were stained with Relaxin-2 antibody, and the amount of staining for relaxin receptors was determined using an ordinal H score.

**Results:**

The mean (SD) H scores of infants with DDH were significantly higher than those of controls: 215 (59) versus 52 (48); *P* = 0.00; 95% CI. Statistically significant difference between the two groups in terms of gender was not found.

**Conclusion:**

As a result, increased number of relaxin receptors in the ligamentum teres could be a risk factor for DDH.

**Level of evidence:**

Level 2, Prospective comparative study.

## Introduction

The incidence of developmental dysplasia of the hip (DDH) is 2.7 to 17 per 1000 births worldwide [1].

Hormonal changes, as well as genetic diseases such as Ehler–Danlos syndrome, may be involved in DDH, which is associated with joint laxity [2]. The relaxin hormone, which is necessary for the preparation of the maternal pelvis for labor and pelvic relaxation, passes to the infants through the placenta and causes laxity. In addition, the fact that girls are more sensitive to this hormone explains the higher DDH rates in girls compared to boys and it is one of most common hormones associated with DDH [3]. Relaxin has collagenolytic effects on ligamentous tissue in both pregnant and non-pregnant women. It may also be associated with laxity of fetal ligaments [4]. Increased serum relaxin concentration may result in increased joint laxity, as shown by Steinetz et al. in 2008. In the study, they reported that the relaxin levels are increased in pregnant women with pelvic join instability or hip joint laxity compared with the control group [5]. 3 years later Dragoo et al. reported that increased serum relaxin levels resulted in reduced ligament integrity and increased risk of injury for anterior cruciate ligament (ACL) [6].

Two hypotheses address the importance of relaxin in joint laxity**.** One hypothesis is that relaxin exerts a direct effect on fetal ligament laxity by mediating progesterone and estrogen concentrations in connective tissue [7]. Relaxin also stimulates collagen turnover by increasing the collagenase expression and down-regulates collagen synthesis [8]. The second hypothesis is that it exerts an indirect effect that reduces relaxin concentrations in maternal blood. This reduction can impair relaxation of the pelvic ligaments and thus lead to malpositioning the fetus [9]. However, relaxin's effect on joints as mediated through weakened ligaments remains unclear. Although prolonged exposure to relaxin during pregnancy may increase joint laxity, acute effects of short-term relaxin exposure on ligaments has not been determined [10].

The ligament of head of femur is extending between the femoral head and the acetabulum. It is a collagenous structure more robust than the capsule in fetuses and may be important in stabilizing the fetal hip joint [11]. The ligament may prevent or slow the progression of an unstable hip to a dislocated hip, particularly at term, when the discrepancy between the size of the femoral head and socket depth is greatest [12]. In fact, Weinstein and Ponseti reported that the ligamentum teres in patients with DDH is longer and thicker than normal, as a result of laxity and the abnormal position of the femoral head [13].

We believe that higher relaxin concentrations in patients with DDH may lead to pathodynamic changes during hip development by altering the physiological nature of the ligament, as well as by long-term exposure to relaxin during pregnancy. Our objective in this study was to determine the quantity of relaxin receptors in the ligamentum teres and their part in causing DDH.

## Patients and methods

This prospective study was approved by the institutional review board of authors’ affiliated institutions. Parents and guardians provided written informed consent to have their children enroll in the study and to have samples taken for analysis.

We identified 26 infants between birth and 3 years of age who had undergone open reduction for DDH between 2010 and 2012. 12 hips of 12 miss abortus fetus between 20 to 35 weeks of gestation were used as control group. Infants with teratologic hip dislocations, any accompanying congenital syndrome or a history of intra-articular intervention of the hip, such as hip surgery or arthrography, were excluded.

### Immunohistochemical analysis

**Fig. 1:**
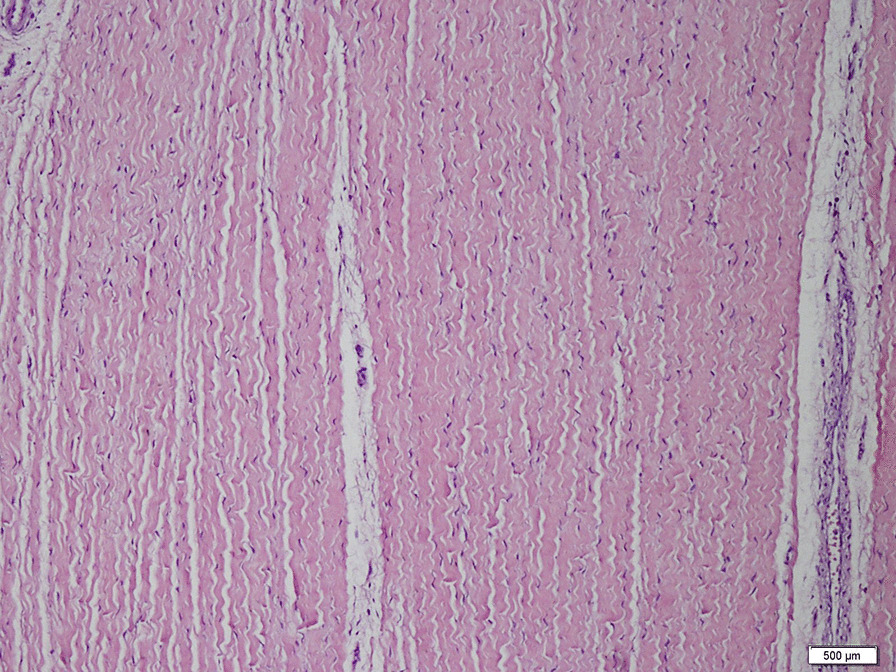
Morphology of the ligamentum teres. Note the collagen bundles extending parallel to each other (Hematoxilen & eosin;X100)

The ligamentum teres ligaments were harvested from infants who had undergone open reduction through a medial or anterolateral incision, fixed in 10% formaldehyde for 24 h, and embedded in paraffin blocks. Sections, 3-µm thick, were stained with hematoxylin and eosin. Sections were mounted on lysine-coated slides and deparaffinized for immunohistochemical analysis (Fig. 1).

**Fig. 2:**
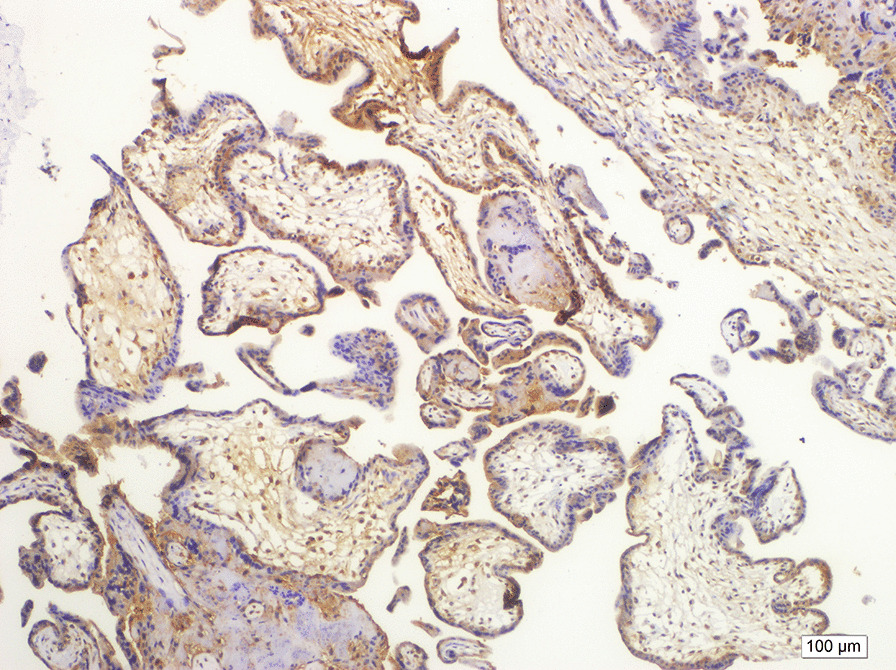
Placental tissue as a positive control for relaxin. Brown cytoplasmic staining was accepted as positive staining. (Hematoxilen & eosin; X200)

**Fig. 3:**
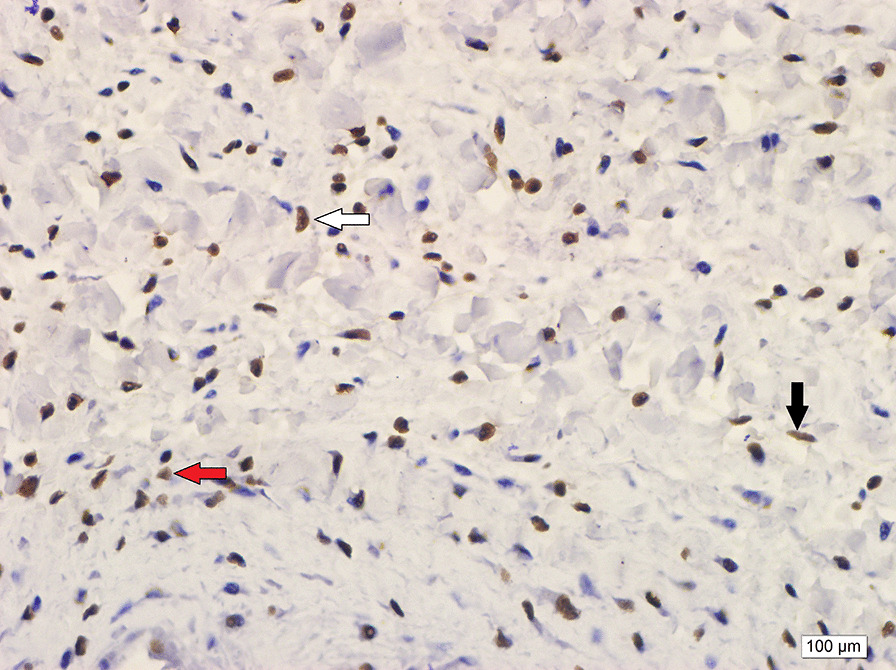
Examples of staining intensity of relaxin. White arrow, example of a 3+ result; black arrow, 2+; and red arrow, 1+ stained cells (Hematoxilen & eosin; X200)

Antigen retrieval was performed in a citrate buffer solution, and relaxin-2 antibody (GPR106 antibody, Biorbyt, US) was used at 1:50 dilution. Placental tissue was used as a positive control (Fig. 2). We stained the nucleus of cells with relaxin antibody. Preparations were analyzed under light microscopy at a magnification of 400× at the most intense staining area. The intensity of staining for relaxin was categorized as low, medium, and high, as indicated in Fig. 3. The proportion of cells in each staining category was determined from 100 cells in the densest area of staining by a single pathologist with experience in immunologic staining. Pathologist was blinded for cases and control groups.

The amount of staining for relaxin receptors was determined using an ordinal H score [14]

H score = 3 × (percent of cells stained at high intensity) + 2 × (percent of cells stained at medium intensity) + 1 × (percentage of cells stained at mild intensity). The H scores range from zero (no relaxin receptors) to 300 (all cells are stained with relaxin at high intensity). They are significantly correlated with the PCR data and are also sensitive to small changes in receptor expression [15].

### Statistical methods

The H scores were compared between groups with two-tailed Student's *t*-test. Alpha was set at 0.05. Data were analyzed with the SPSS software package.

## Results

We analyzed data from 26 infants (20 female) who underwent open reduction for DDH and 12 hips of 12 fetus (9 female) used as control group. The 26 infants ranged in age from 10 to 36 months (mean [SD] age, 22 [6] months).

The mean (SD) H scores of infants with DDH were significantly higher than those of controls: 215 (59) versus 52 (48); *P* = 0.00; 95% CI, (Table 1). Statistically significant difference between the two groups in terms of gender was not found.

**Table 1:** Results of Staining the Ligamentum Teres for Relaxin Receptors in Infants with Developmental Dysplasia of the Hip Treated with Open Reduction and Control Infants with Hip Fractures Treated with Hip Replacement

Characteristic	Infants with developmental dysplasia of the hip, *n* = 26	Missed abortus fetuses as control *n* = 12	*P*
Age, mean (SD), months	21.7 (5.74)	27.8 (5.4) weeks	
Females, *n* (%)	20 (77%)	9 (75%)	> 0.05
Right hip involved, *n* (%)	10 (38%)	5 (42%)	> 0.05
H score^a^, mean (SD)	215 (59)	52 (48)	< 0.001

^**a**^H score = 3 × (percent of cells stained at high intensity) + 2 × [(percent of cells stained at medium intensity) + 1 × (percentage of cells stained at mild intensity)]. See text for details

There was no statistically significant difference between the age at the time of operation time and H scores of infants (*p* < 0.05).

## Discussion

The cause of developmental dysplasia of the hip is still debated, although ligamentous laxity is a contributing factor [1]. Few studies have found evidence for a hormonal effect on DDH. Only a few studies have found an association between DDH and serum or cord blood relaxin concentrations, and many others have found no association. However, our study appears to be the only one that measured relaxin receptors in the ligament of head of femur [16].

Relaxin acts on the extracellular matrix through metalloproteinases by enhancing collagen degradation [8]. Relaxin is associated with both local and generalized laxity [13]. In patients with laxity-associated arthrosis of the first carpometacarpal joint, the concentration of relaxin inhibitors was higher in the volar oblique and anterior oblique ligaments, indicating that relaxin was involved as a cause of laxity [17, 18]. Another study in healthy humans found that trapezial-metacarpal joint laxity was proportional to serum relaxin concentrations [13]. A study in rats found that recombinant human relaxin 30 ng/mL weakened the joint capsule and decreased collagen accumulation in a dose-dependent way [19].

In women athletes, the “monthly window of potential injury” has been associated with hormonal changes, as defined by Möller-Nielsen [20]. This window coincided with peak concentrations of relaxin during the menstrual cycle, and generalized joint laxity was increased during these peak times [20]. Relaxin also increased ligamentous laxity during pregnancy [21]. Relaxin receptors are up regulated by estrogen therapy, and joint laxity occurring after the first trimester of pregnancy is synchronous with peak relaxin contractions [21].

Steinetz et al. hypothesized that increased serum relaxin concentration may result in increased joint laxity. In their study, they reported that increased levels of relaxin in pregnant woman with pelvic join instability or hip joint laxity compared with the control group. (Y) 3 years later, Dragoo et al. reported that increased level of serum relaxin resulted in reduced ligament integrity and increased risk of injury for anterior cruciate ligament (ACL) (Y-1).

In one of two other separate studies by Dragoo, tibial translation in guinea pigs was increased by 12.8% (absolute increase, 1.09 mm) 21 days after relaxin administration. The maximum load of the anterior cruciate ligament was also weaker by 36%, dropping from 64.1 to 40.4 N, and relaxin altered the structure of the ligament by collagenolytic effects [22]. In the second study, relaxin increased the synthesis of collagenase and many matrix metalloproteinases in anterior cruciate ligament rupture and decreased local collagen synthesis in women orthopedic patients [23]. Increases in estrogen and relaxin during the normal menstrual cycle last only a very short time and are insufficient to explain any collagenolytic effect. It appears that long-term fluctuations are required to remodel the collagen [23].

Prolidase activity is increased in patients with DDH [24]. High prolidase activity indicates increased collagen metabolism, which is consistent with relaxin's mechanism of action. In the present study, we found that the number of relaxin receptors was twice as high in infants with DDH than in the elderly control group. We believe that this higher number of receptors and long-term exposure to relaxin from the mother during pregnancy may alter the nature of the ligamentum teres, resulting in hyperlaxity, possibly predisposing the fetus to DDH. However, in this case, it appears that the higher number of fetal receptors, rather than the concentration of relaxin from the mother, might be the cause because Forst et al. found that relaxin concentrations in specimens from cord blood were lower in neonates with DDH than in those of a healthy control group [4]. In a similar study, Vogel et al. also found no relationship between relaxin concentrations and DDH in 15 infants with DDH [25].

The ligamentum teres is important in stabilizing the pathophysiology of DDH [26]. Wenger et al. suggested that the strength of the ligamentum teres was crucial in the biomechanics of the hip and recommended surgical reconstruction for patients with DDH [26]. Another study on rats reported that greater strength of the ligamentum teres helps proper remodeling of the acetabulum against the femoral head [27].

The risk of DDH is four times as high in females as in males [28]. Desteli et al. found a negative relationship between the number of estrogen receptors and collagen metabolism in the ligamentum teres harvested from infants with DDH [29]. Furthermore, relaxin receptor isoforms are down-regulated with progesterone and high-dose estrogen, increasing the laxity, whereas progesterone and estrogen are down-regulated with testosterone [30]. Testosterone concentrations are 8 times as high in men as they are in women, and testosterone reduces the expression of the relaxin receptor, as well as decreasing the passive range of motion in joints [31].

The following questions arise from these findings: because relaxin is a maternal hormone, is it likely that attenuated relaxin activity improves a hip from Graf type IIa to type I in neonates? In addition, in hips with more relaxin receptors, although relaxin concentrations are reduced in time, does the long-term action of relaxin cause permanent dysplasia? Finally, can the down-regulation of relaxin receptors caused by testosterone in men be the reason for their lower rate of DDH? All these questions may guide future studies. In this preliminary study, we used a single evaluator to score the intensity of staining. Given that we found an association between relaxin receptors and DDH, however, future studies should reduce the chances of bias by using two or more evaluators.

Our study has some limitations. First, our study group and control group have a significant age difference which may influence serum relaxin levels and intraligamentous relaxin levels. Our study group consists of babies less then 3 months old, but our control group were miss abortus fetuses between 20 and 35 weeks of gestation. Second, as the hip development finishes before 20 weeks, we used to prefer fetuses over 20 weeks, but we cannot be sure that these fetuses have a genetic abnormality or would develop a DDH in future. Third, our sample size is not as big as it should be for assessing a certain factor playing a role in developmental hip dysplasia.

## Conclusions

Our data support the conclusion that the number of relaxin receptors in the ligamentum teres is significantly higher in infants with DDH than in healthy control patients and increased number of relaxin receptors in the ligamentum teres could be a risk factor for DDH.

## Data Availability

Datasets are available in the form of an excel table.

## Abbreviations

DDH: developmental dysplasia of hip
ACL: anterior cruciate ligament
